# Evaluation of pathogenic serovars of *Leptospira interrogans* in dairy cattle herds of Shahrekord by PCR

**Published:** 2011-09

**Authors:** A Jafari Dehkordi, HR Shahbazkia, N Ronagh

**Affiliations:** 1 Departments of Large Animal Internal Medicine; 2 Biochemistry Veterinary Faculty, Shahrekord University, Iran; 3 Veterinary Faculty, Shahrekord University, Shahrekord, Iran.

**Keywords:** *Leptospira*, Shahrekord, Cattle, PCR

## Abstract

**Background and objectives:**

Leptospirosis is an important zoonotic disease caused by *Leptospira interrogans*. Leptospirosis leads to economical losses in dairy farm industry. The objective of this study was to evaluate the pathogenic serovars of *Leptospira interrogans* in dairy cattle herds of Shahrekord by PCR.

**Materials and Methods:**

Two hundred samples (100 urine and 100 blood) were collected from 100 cows randomly and delivered to the laboratory. Samples were stored at -20 °C. DNA was extracted and purified from the plasma and urine samples and concentrated on diatoms in the presence of guanidine thiocyanate (GuSCN). PCR products were detected and identified as *Leptospira* by ilumination of the expected size of DNA bands after staining of the agarose gel with ethidium bromide gels. PCR products were purified and sequenced.

**Results:**

The results showed that 28% of urine samples and 23% of plasma samples were contaminated. The major serotypes were *Icterohaemorrhagiae* (50%) and *Pomona* (37.5%). The urine samples of 17 cows were positive for *Leptospira* without positive plasma samples. This indicated that these cows are reservoirs in dairy herds of Shahrekord and dangerous for human health. The plasma samples of twelve cows were positive for *Leptospira* without positive urine samples.

**Conclusions:**

*Leptospira* serotypes can be maintained in relatively dry regions and must be considered when dealing with leptospirosis in dairy farms of Shahrekord and human health.

## INTRODUCTION

In the recent years, leptospirosis is identified as a global public health problem because of its increased mortality and morbidity in different countries (1,2). Leptospirosis is caused by pathogenic spirochaetes of the genus Leptospira. The organism affects many mammalian species, including humans. Animals may become inapparent carriers and shedders of leptospires, primarily in the urine, serves as a source of infection for other animals and humans (3). In cattle, leptospirosis is an important cause of abortion, stillbirths, infertility, poor milk production and death, all of which cause an economic loss (4). The bacteria can survive in damp soil, fresh water, mud, and vegetation for a long time. Hence, the mode of transmission in human is either by contact with contaminated soil or water or with body fluid of infected animals and may lead to potential lethal disease (2,5). Members of the genus *Leptospira* are conventionally grouped into 2 separate species based on pathogenicity. The pathogens are from the parasitic “interrogans” group, whereas the nonpathogens are from the saprophytic “biflexa” group. Normally, Leptospira interrogans but not Leptospira biflexa can be isolated from the patient's blood, urine, and cerebrospinal fluid. However, epidemiologic studies may require samples to be taken from fresh surface water of lakes or streams where L. interrogans and L. biflexa species can coexist (6,7).

Laboratory diagnosis of leptospirosis is a confusing topic for treatment and surveillance because of its varied symptoms. In addition, delay in treatment of patients, due to the lack of available effective techniques for rapid diagnosis of disease may cause lethal sequel (8,9).

The clinical signs associated with bovine leptospirosis are variable and depend on the infecting serovar and the susceptibility of the animal. Clinically, bovine leptospirosis is difficult to diagnose because the signs are non-specific and easily confused with other diseases (4). Traditionally, the reference method for diagnosis of leptospirosis is the microscopic agglutination test (MAT). However, this test has several drawbacks, including the requirement for a permanent stock of reference strains representing the appropriate serogroups, subjectivity involved in reading the results under dark-field microscopy, inability to differentiate titers of natural infection from vaccinal titers and the failure to identify most chronic shedders (10). Moreover, the assay is labour intensive and represents a biohazard to laboratory staff (3, 11–(13). Isolation of leptospires is time consuming, subject to contamination and may require 4–6 months (4). A variety of molecular methods have been developed for the specific detection of pathogenic Leptospira spp. serovars in clinical samples. These include DNA–DNA hybridization (14), in situ hybridization (15) and DNA probes (16), which have been used mainly for detection of leptospires in urine samples from animals infected experimentally with Leptospira. The polymerase chain reaction (PCR) also has been used to detect Leptospira spp. in urine samples from cattle experimentally infected with serovars Leptospira (17–21). A PCR to detect Leptospira spp. in the urine of naturally infected cattle using genus-specific primers has been reported (22). Recently, a nested PCR with primers derived from the LipL32 sequence has been reported by Nassi et al (23) and Jouglard et al (13) to detect Leptospira spp. from clinical samples including urine and serum. Since leptospirosis is dangerous to humans and the climate of Shahrekord is not suitable for Leptospira, the aim of this study was to detect pathogenic serovars of Leptospira interrogans from dairy cattle in Shahrekord by PCR and to trace the carrier animals.

## MATERIALS AND METHODS

In this study, 100 blood samples (via jugular vein) and 100 urine samples (via urinary catheter) were collected (200 samples) from 100 cows randomly. Samples were stored at -20°C, and delivered to Shahrekord University laboratory.

DNA was extracted and purified from the plasma and urine samples and concentrated on diatoms in the presence of guanidine thiocyanate (GuSCN). Briefly, 100 l of plasma or urine were added to 900 l of L6 buffer (GuSCN 120 g, 0.1 M Tris-HC1, pH 6.4, 100 ml, 0.2 M EDTA 22 ml, Triton X-100 2.6 ml) with 40 l of diatom suspension (diatoms 10 g, distilled water 50 ml, 500 l of HCl 36 YO w/v). The mixture was vortexed and incubated at room temperature for 10min and then centrifuged to spin down the complex of DNA-diatoms. After washing twice with L2 buffer (GuSCN 120 g, 0.1 M Tris-HCL 100 ml, pH 6.4), twice with ethanol 70% v/v, and once with acetone, the DNA-diatom complex was dried at 56°C for 10min and the DNA was eluted in the presence of proteinase IS 120 pg/ml solution at 56°C for 10 min. The proteinase K was subsequently inactivated by incubation at 100°C for 10 min (24).

Primers Gl(5’ CTG AAT CGC TGT ATA AAA GT) and G2 (5’ GGA AAA CAA ATG GTC GGA AG) were derived from the 5’ end (nucleotides 1-20) and the 3’ end, of the sequence of the recombinant plasmid pLIPs60 (nucleotides 264-285) respectively. Primers B64-I (5’ CTG AAT TCT CAT CTC AAC TC) and B64-I1 (5’ GCA GAA ATC AGA TGG ACG AT) were derived from the 5’ end (nucleotides 1-20) and the 3’ end of the sequence of recombinant plasmid pBIM64 (nucleotides 542-563) respectively, (24).

PCR was performed as described previously with minor modifications (24). Briefly, 40 l of DNA samples were mixed with 5 l of the reaction buffer (10 x buffer: 500 mM-KC1, 20 mMMgCl, 100 mM-Tris/HCl, pH 9.0, 0.5 l of a 100 PM solution of each primer, 0.5 l of a mixture containing 25 mM of each of the four deoxynucleotides dATP, dTTP, dCTP and dGTP, 0.1 l Taq polymerase (0.5 U) and 3.4 l distilled water to a final volume of 50 l, DNA amplification reactions were performed in a Biorad thermal cycler using 32 cycles. One amplification cycle consisted of denaturation of the DNA for 90 s at 94 “C, annealing of the primers for 60 s at 55 “C and elongation for 120 s at 72 “C (24). PCR amplification products were detected and identified as Leptospira-specific DNA by illumination of agarose gel after electrophoresis and staining with ethidium bromide. A patient was scored positive if either the plasma or urine sample gave a positive PCR result, i.e., a 285-bp fragment with primers G1/G2 or a 563-bp fragment with primers B64-I/B64-11, accompanied by corresponding hybridisation with the labelled probes. The preparation of reaction mixtures, the DNA extraction (clinical samples and positive controls) and the subsequent amplification and detection of the PCR products were all performed at different locations within one building. This strict spatial partition of the different technical steps involved in the PCR was necessary to prevent contamination. In addition, tables and equipment were decontaminated periodically with HCl 10%.

PCR products were purified and sequenced in an applied Biosystems 3730xl Automatic DNA Sequencer by Macrogen (Korea) using amplification primers. The partial sequences of the VNTR loci of the field strains of this study and the intrrogans reference strain have been deposited in GenBank under the Accession Numbers GU362888–GU362928.

DNA sequences were analyzed using the GenBank database of the National Center for Biotechnology Information BLAST network service. Tandem Repeats Finder program was used to define exactly the copy number of each VNTR locus (25). UPGMA (unweighted pair group method with arithmetic mean) clustering analysis was performed using the Sequence Type Analysis and Recombinational Tests (STAR) software on genotype scores (26).

## RESULTS

The results of this study showed that 28% of urine samples and 23% of plasma samples were contaminated. The major serotypes were Icterohaemorrhagiae (50%) and Pomona (37.5%). It should be noted that 17 urine samples have negative plasma samples. This indicated that cows are reservoir in dairy herds of Shahrekord. Twelve plasma samples without having positive urine samples were estimated as positive for Leptospira.

**Fig. 1:**
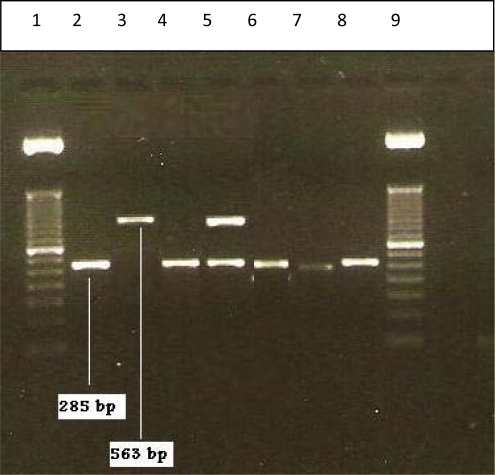
Agarose gel electrophoresis of amplified DNA from plasma and urine samples using primers G1/G2 (285-bp product) and B64-I/B64-11 (563-bp product). Lane 1 and 9, molecular weight marker VIII (Boehringer Mannheim); 2, DNA from interrogans amplified with G1/G2; 3, DNA from bim amplified with B64-I/B64-11; 3 DNA from Kirschneri amplified with B64-I/B64-11, Lane 4 Gl/G2; Lane 5 Gl/G2 and B64-I/B64-11, Lane 4 Gl/G2, Lane 7 doubtful, Lane 8 Gl/G2.

All samples were tested at least twice by PCR and gave reproducible results. The amplicons obtained from PCR-positive samples were visualized on agarose gel electrophoresis (Fig. 1).

## DISCUSSION

The results of this study show that the urine samples in 17% of cows served as a reservoir of disease in dairy farms of Shahrekod district while they were negative in their plasma samples. So it could be stated that the animal reservoirs increase the risk of potential spread of disease to other animals and especially humans, and this deserves special attention.

Production of antibodies against Leptospira in the body occurs several days after the occurrence of Leptospiremy and rapidly starts clearing bacteria from blood and tissue. Some of the leptospiras usually can be reached out of the immune system and may persist in kidney tubules, liver, uterus, eye and meninge. Urease enzyme production is a factor for durability of the bacteria in the kidneys. Animals that recovered from acute leptospirosis may be carrying the disease and leptospiras remain in their kidney tubules from a few days to several years, although in these cases, the agent is not found in blood but is excreted through urine (27). So, in this study 17% of urine samples were estimated as a reservoir of disease in dairy farms of Shahrekod district without having positive plasma samples.

Also, in this study 12% of plasma samples were estimated positive for Leptospira without positive urine samples. Probably these animals were in the early stages of the disease, and their immune system still did not completely remove bacteria from the blood. Bacteria may not have found sufficient time for colonization in the kidney and this resulted in negative urine samples. Also, it should be considered that some serovar of Leptospira are frequently excreted through the urine, and this may be why it is free of Leptospira at the time of sampling in this study (1).

In a study conducted in Shahrekord district in 1997, 19% of the samples from 100 cows of 8 dairy farms were positive for Leptospira. The highest and lowest serovar contamination was Icterohaemorrhagiae (36.8%) and serovar Canicola (10.5%) respectively (28).

Using MAT method, Ebrahimi et al (2004) found 18.75% of the sera samples collected from 400 cattle of both traditional and industrial dairy farms in Shahrekord district were positive for Leptospira. The highest and lowest prevalence of serovar was Canicola (50.6%) and Pomona (4%) respectively. In this study the high prevalence of Canicola were related to keeping dogs on dairy farms (9).

In the present study, the dominant serovars were Grippotyphosa and Pomona, that primary are hosted by mice. Thus, rodents must be controlled in dairy farms for decreasing prevalence of disease in Shahrekord district.

In studies from 1997 to 2002 conducted in Shahrekord, the predominant serovars had changed from Icterohaemorrhagiae to Canicola. But in the present study (2010), Icterohaemorrhagiae was identified as the prevalent serovar in this region. This indicated that the predominant serovars can be changed in the regions over the time.

Rodrigues et al. (1999), found that Icterohaemorrhagiae and Pomona as dominant serovars in Brazilian cattle during 1996 and 1997. While previous studies had shown that Hardjo and Pomona serotypes were predominant (29). These results suggest that changes in the common serovars in the region occurred.

According to studies conducted in Ahvaz, the high incidence of leptospirosis attributed to hot and humid weather of Khuzestan region and the heat temperature was reported more important than moisture (30). Due to the global warming of the earth, increases in prevalence of the disease over the time can be expected.

According to the provincial weather reports, the annual rainfall during 2001 to 2010 has been constantly fluctuating from 336.8 to 414 mm in Shahrekord. Since serovar Pomona is related to annual rainfall, we conclude that increase in rainfall in Shahrekord is a reason for higher prevalence of Pomona in this study. In a study by Durham and colleagues during 1991-1992 have been done in Australia, Tarassovi and Hardjo serovars, respectively, were having the highest and lowest prevalence and none of this samples did not show positive reaction against Pomona, due to know low rainfall in the area. Because rainfall is very involve in serovar prevalence (30).

Canicola and hardjo serovars were found in Gilan and Ahvaz (30) but in our study three serovars were found in the Shahrekord (Icterohaemorrhagiae, Pomona, Grippotyphosa).

In conclusion, although the disease is seen in tropical countries, it could also be present in cold and mountainous regions such as Shahrekord. Considering the results of this study it should be noted that serovar changes is most common and related to weather condition. So, it is necessary to screen the serovars in every region regularly to prevent the spread of the disease.
